# Comprehensive evaluation of three-dimensional anatomy of perigastric vessels using enhanced multidetector-row computed tomography

**DOI:** 10.1186/s12893-022-01836-0

**Published:** 2022-11-21

**Authors:** Ichirota Iino, Hirotoshi Kikuchi, Toshiyuki Suzuki, Toshiki Kawabata, Osamu Jindo, Akihiro Uno, Atsuko Fukazawa, Keigo Matsumoto, Hideto Ochiai, Takanori Sakaguchi, Hiroya Takeuchi, Hiroyuki Konno, Shohachi Suzuki

**Affiliations:** 1 Department of Surgery, Iwata City Hospital, 512-3 Okubo, Iwata, 438-8550 Japan; 2 Department of Surgery, Hamamatsu University School of Medicine, 1-20-1 Handayama, Higashi-ku, Hamamatsu, 431-3192 Japan; 3 Department of Radiological Technology, Iwata City Hospital, 512-3 Okubo, Iwata, 438-8550 Japan; 4 Hamamatsu University School of Medicine, 1-20-1 Handayama, Higashi-ku, Hamamatsu, 431-3192 Japan

**Keywords:** Laparoscopic gastrectomy, Perigastric vessels, Vessel anatomy, 3D angiography

## Abstract

**Background:**

To perform laparoscopic gastrectomy safely, we aimed to comprehensively re-evaluate perigastric vessel anatomies using a three-dimensional angiography reconstructed from enhanced multidetector-row computed tomography data.

**Methods:**

Perigastric vessel anatomy was preoperatively analyzed using a multidetector-row computed tomography-based three-dimensional angiography reconstructed in 127 patients undergoing gastric surgery.

**Results:**

Of the 67 left gastric veins that ran along the dorsal side of the arteries, 59 (88.1%) ran along the dorsal side of the common hepatic artery and flowed into the portal vein. In 18 cases, a common trunk of one to three left gastric arteries and the replaced left hepatic artery was observed. The left inferior phrenic artery ramified from the left gastric artery in 5.5% of the cases. The right gastric artery was classified into distal (73.2%), caudal (18.1%), and proximal (8.7%) types. The infra-pyloric artery was also classified into distal (64.6%), caudal (26.0%), and proximal (9.4%) types. The posterior gastric artery branched as a common trunk with the superior polar artery in the proximal (37.9%) and distal (18.4%) regions of the splenic artery. The left gastroepiploic artery ramified from the splenic (18.1%) and inferior terminal arteries (81.9%). No, one, and two gastric branches of the left gastroepiploic artery, which ramified between the roots of the left gastroepiploic artery and its omental branch, were found in 36.5%, 49.2%, and 14.3% of the cases, respectively.

**Conclusions:**

Preoperative 3D angiography is useful for the precise evaluation of perigastric vessel anatomies, and may help us to perform laparoscopic gastrectomy and robotic surgery safely.

**Supplementary Information:**

The online version contains supplementary material available at 10.1186/s12893-022-01836-0.

## Background

Gastric cancer is the fifth most common malignancy worldwide. It is one of the major causes of mortality in Central Asia, Eastern Europe, and Eastern/Southeastern Asia, including Japan [1]. In the era of multidisciplinary therapy with advanced chemotherapy and immunotherapy for advanced diseases, gastrectomy with lymphadenectomy plays important roles in the treatment strategies for early and advanced gastric cancer [2]. Laparoscopic gastrectomy has been gradually accepted in recent years because of its advantages over open surgery, including minimal invasiveness, less pain, and lower rate of overall complications, as well as its non-inferiority to open gastrectomy for survival [3–7]. Although laparoscopic gastrectomy is minimally invasive and enables precise manipulation with enlarged visual field, some technical limitations apply for more advanced surgical procedures or for the treatment of locally advanced cancers. As the indication of laparoscopic gastrectomy has gradually increased in recent years, the importance of preoperative evaluation of perigastric vascular anatomy has also increased [8]. However, comprehensive analyses of perigastric vascular anatomy have not been well conducted.

Recent advances in three-dimensional (3D) computed tomography have enabled the examination of vascular anatomy without percutaneous catheter angiography. Previous studies reported the usefulness of 3D angiographic analysis of perigastric vessels [9–15]. In contrast to conventional angiography, multidetector-row computed tomography (MDCT)-based 3D angiography enables angle-free observations to be performed. In our earlier report, we classified the ramification pattern of the right gastric artery (RGA) into distal, caudal, and proximal types. We also showed that RGA ramification points can be misdiagnosed under conventional angiographic anterior views because of the lack of 3D information [15]. Therefore, perigastric vascular anatomy should be re-evaluated using 3D angiography in the era of laparoscopic surgery.

In this study, we aimed to comprehensively evaluate perigastric vascular anatomy using a 3D angiography reconstructed from enhanced MDCT data and to discuss the usefulness of preoperative 3D angiography from the perspective of laparoscopic gastrectomy.

## Methods

### Patients

This study retrospectively enrolled 127 consecutive patients who underwent MDCT followed by gastrectomy between August 2015 and July 2018 at Iwata City Hospital. The patient population consisted of 91 men and 36 women aged 37 to 86 years (median age: 70 years). A total of 121 patients had gastric cancers, and 6 had gastric gastrointestinal stromal tumors.

### Computed tomography protocol

The protocol was described in our previous report [15]. Images were obtained using the 320-detector row computed tomography (CT) scanner (Aquilion ONE/ViSION Edition; Canon Medical Systems, Otawara, Japan) or Brilliance iCT (Philips Healthcare, Cleveland, OH, USA). A 20-G intravenous catheter was inserted into the medial cubital vein. The range of contrast-enhanced CT scans was set to cover the area from the dome of the liver to the aortic bifurcation. With contrast-enhanced CT images, a nonionic contrast agent (370 mg or 300 mg I/mL, Omnipaque; Daiichi Pharmaceutical, Tokyo, Japan) was infused rapidly at 40 mg I/kg for 25 s with an automated injector. A bolus tracking method was performed to obtain early arterial phase images. Early arterial phase scanning initiated when Hounsfield units reached 200 in the abdominal aorta at the bifurcation level of the celiac artery (CA). The average scanning delay between the start of contrast material injection and the start of early arterial phase scanning was 20 s (range: 15–28 s). Late arterial phase scanning and early venous phase scanning were initiated 10 s and 30 s after early arterial phase scanning, respectively.

### 3D angiography by workstation

Volume data were transferred to a workstation (SYNAPSE VINCENT; Fujifilm Medical, Tokyo, Japan). Arteriography was obtained from the early arterial phase scanning data. Portography was prepared either from the late arterial phase or the early venous phase data. Arteriography and portography were subsequently combined.

## Results

### Branching patterns of the CA and the left gastric artery (LGA)

Branching patterns of the CA and the LGA were classified according to Adachi’s classification [16]: Type I, common trunk of LGA, splenic artery (SA), and common hepatic artery (CHA); Type II, common trunk of SA and CHA; Type III, common trunk of CHA, SA, and the superior mesenteric artery (SMA); Type IV, common trunk of LGA, SA, CHA, and SMA; Type V, common trunk of LGA and SA, and another common trunk of CHA and SMA; and Type VI, common trunk of LGA and SA, and CHA branched from SMA running along the dorsal side of the portal vein. One hundred and eight patients were classified into Type I (85.0%, Additional file 1: Fig. S1a), 7 into Type II (5.5%, Additional file 1: Fig. S1b), 2 into Type III (1.6%, Additional file 1: Fig. S1c), 1 into Type IV (0.8%, Additional file 1: Fig. S1d), and 6 into Type VI (4.7%, Additional file 1: Fig. S1e). Of three cases with miscellaneous branching patterns of the celiac artery, CHA was branched from the aorta and had common trunk of LGA and SA in 2 cases, and LGA was absent but RGA nourished the lesser gastric curvature in the other.

### Running aspects of the left gastric vein (LGV)

**Fig. 1:**
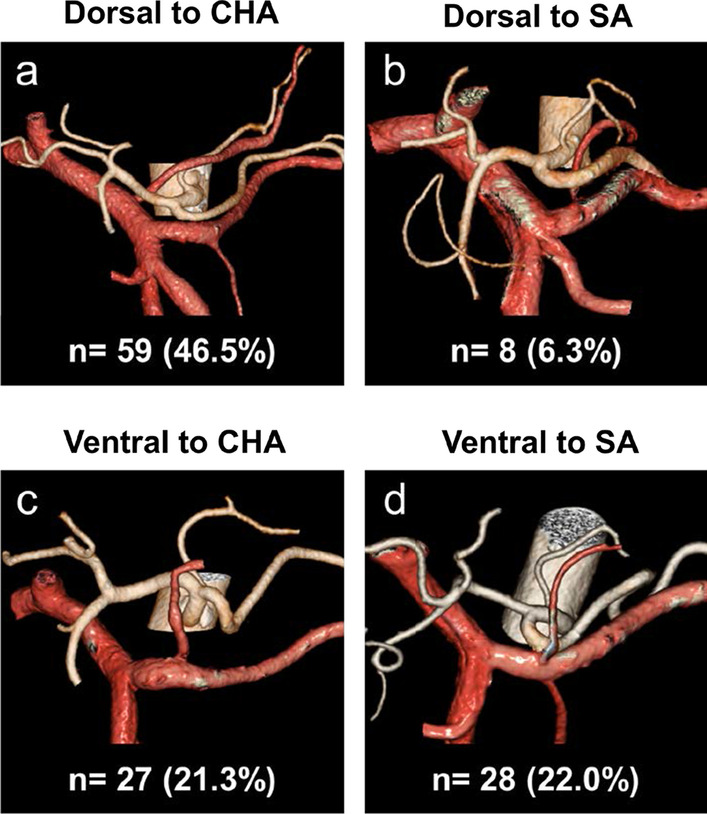
The joining patterns of the left gastric vein (LGV). **a** LGVs running along the dorsal side to the common hepatic artery (CHA). **b** LGVs running along the dorsal side to the splenic artery (SA). **c** LGVs running along the ventral side to the CHA. **d** LGVs running along the ventral side to the SA

The LGV was successfully visualized using 3D angiography in all 127 cases (100%). We classified the running aspects of the LGV into four types: dorsal to the CHA (Fig. 1a), dorsal to the SA (Fig. 1b), ventral to the CHA (Fig. 1c), and ventral to the SA (Fig. 1d). The running aspects of the LGV could be evaluated in 122 cases (96.1%), but those in 5 cases were unclassifiable because of the lack of representative CHA used as a reference artery for LGV classification. In all of 59 cases (46.5%) with an LGV running along the dorsal side to the CHA, the LGV joined the portal vein (PV) that included the splenoportal confluence (Fig. 1a). In all of 8 cases (6.3%) with an LGV running along the dorsal side to the SA, the LGV joined the splenic vein (SV) (Fig. 1b). Of the 27 cases (21.3%) with an LGV running along the ventral side to the CHA (Fig. 1c), the LGV joined the SV and PV in 18 cases (66.7%) and 9 cases (33.4%), respectively. Of the 28 cases (22.0%) with an LGV running along the ventral side to the SA (Fig. 1d), the LGV joined the SV and PV in 26 cases (92.9%) and 2 cases (7.1%), respectively.

### Branching patterns of the LGA from a common trunk with the replaced left hepatic artery (rLHA)

**Fig. 2:**
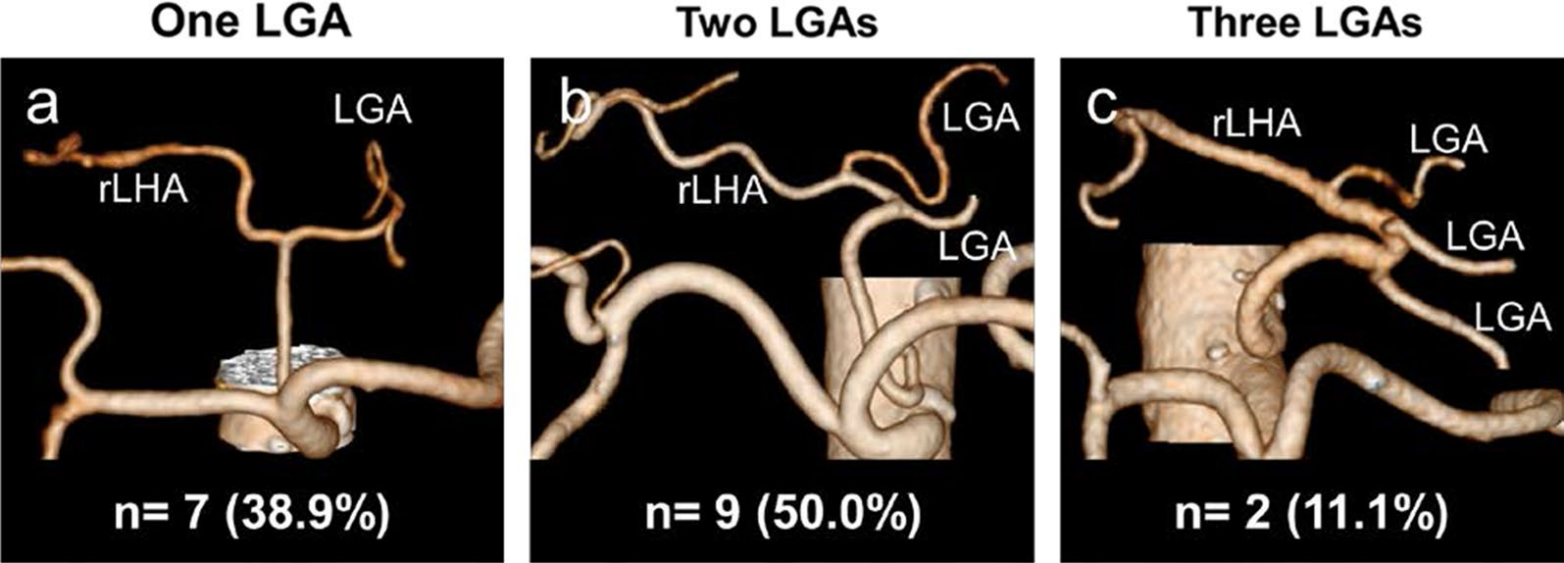
Representative images of 18 cases with a common trunk between the left gastric artery (LGA) and the replaced left hepatic artery (rLHA). **a** There is one LGA branching from a common trunk. **b** There are two LGAs branching from a common trunk. **c** There are three LGAs branching from a common trunk

**Fig. 3:**
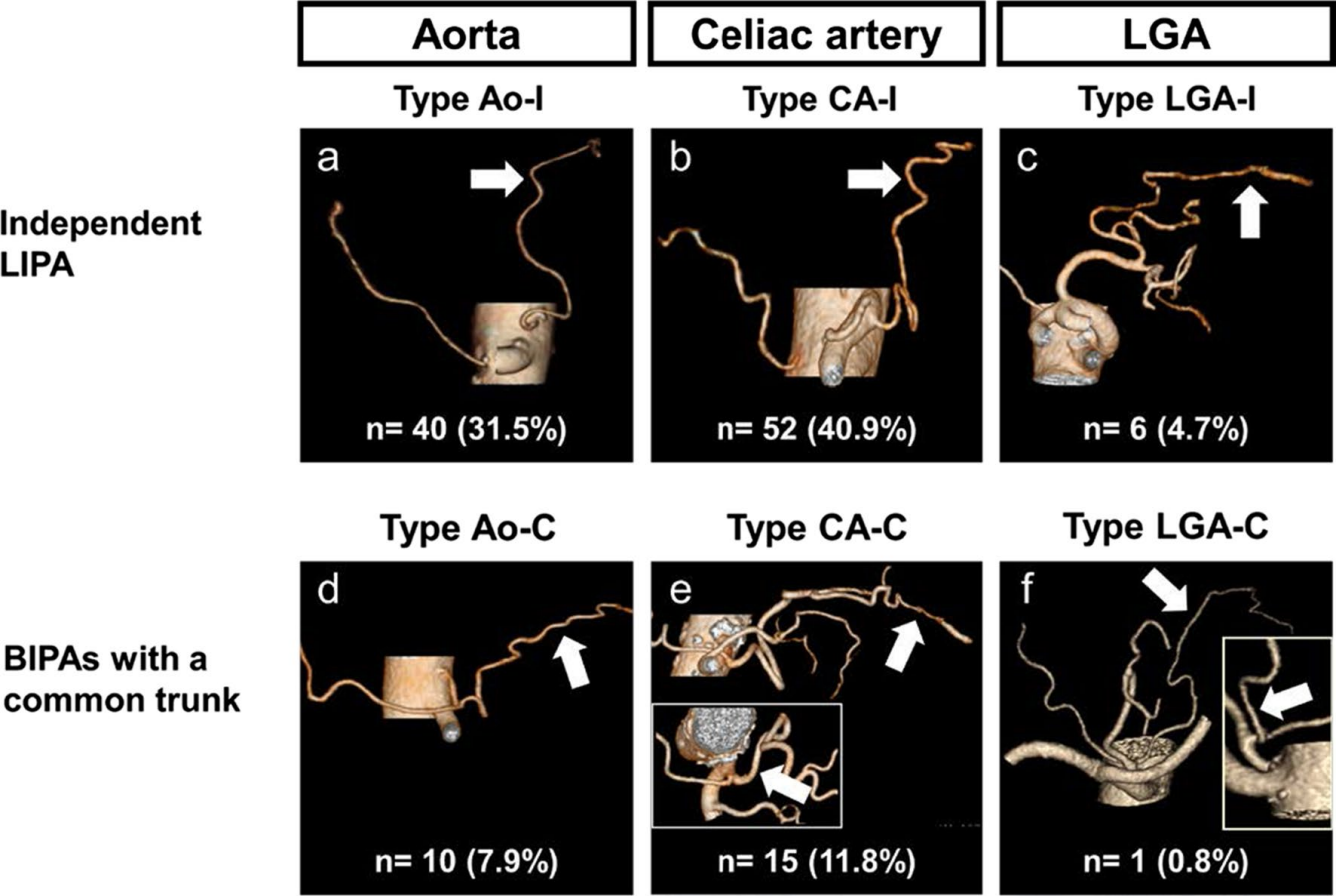
The branching patterns of the left inferior phrenic artery (LIPA). **a** Type Ao-I, the LIPA branching independently from the aorta (Ao). **b** Type CA-I, the LIPA branching independently from the celiac artery (CA). **c** Type LGA-I, the LIPA branching independently from the left gastric artery (LGA). **d** Type Ao-C, the bilateral inferior phrenic arteries (BIPAs) with a common trunk branching from the Ao. **e** Type CA-C, the BIPAs with a common trunk branching from the CA. **f** Type LGA-C, the BIPAs with a common trunk branching from the LGA. *Arrows* indicate the LIPA

The LGA was clearly visualized using 3D angiography in 126 cases (99.2%). There were 24 cases (18.9%) with LGA branches running to the left lobe of the liver including the rLHA and the accessory left hepatic artery. Of these cases, a common trunk with the rLHA was observed in the LGA in 18 cases (14.2%; Fig. 2). Of the 18 cases, 1 LGA branched from a common trunk with the rLHA in 7 cases (38.9%; Fig. 2a), and 2 and 3 LGAs branched from a common trunk in 9 (50.0%; Fig. 2b) and 2 cases (11.1%; Fig. 2c), respectively.

### Branching patterns of the left inferior phrenic artery (LIPA)

The LIPA was clearly visualized using 3D angiography in all 127 cases. The branching patterns of the LIPA were classified based on its branching point: type Ao-I, the LIPA branching independently from the abdominal aorta (Ao); type CA-I, the LIPA branching independently from the CA; type LGA-I, the LIPA branching independently from the LGA; type Ao-C, the bilateral inferior phrenic arteries (BIPAs) with a common trunk branching from the Ao; type CA-C, the BIPAs with a common trunk branching from the CA; and type LGA-C, the BIPAs with a common trunk branching from the LGA (Fig. 3). Forty cases (31.5%) were classified into type Ao-I (Fig. 3a); 52 cases (40.9%), type CA-I (Fig. 3b); 6 cases (4.7%), type LGA-I (Fig. 3c); 10 cases (7.9%), type Ao-C (Fig. 3d); 15 cases (11.8%), type CA-C (Fig. 3e); and 1 case (0.8%), type LGA-C (Fig. 3f). While the branching patterns of the LIPA could be classified in 124 patients, it was unclassifiable in 3 cases. The BIPAs branched from the right renal artery with a common trunk in 1 case, and the LIPA branched independently from the left hepatic artery (LHA) in the others.

### Branching patterns of the RGA

**Fig. 4:**
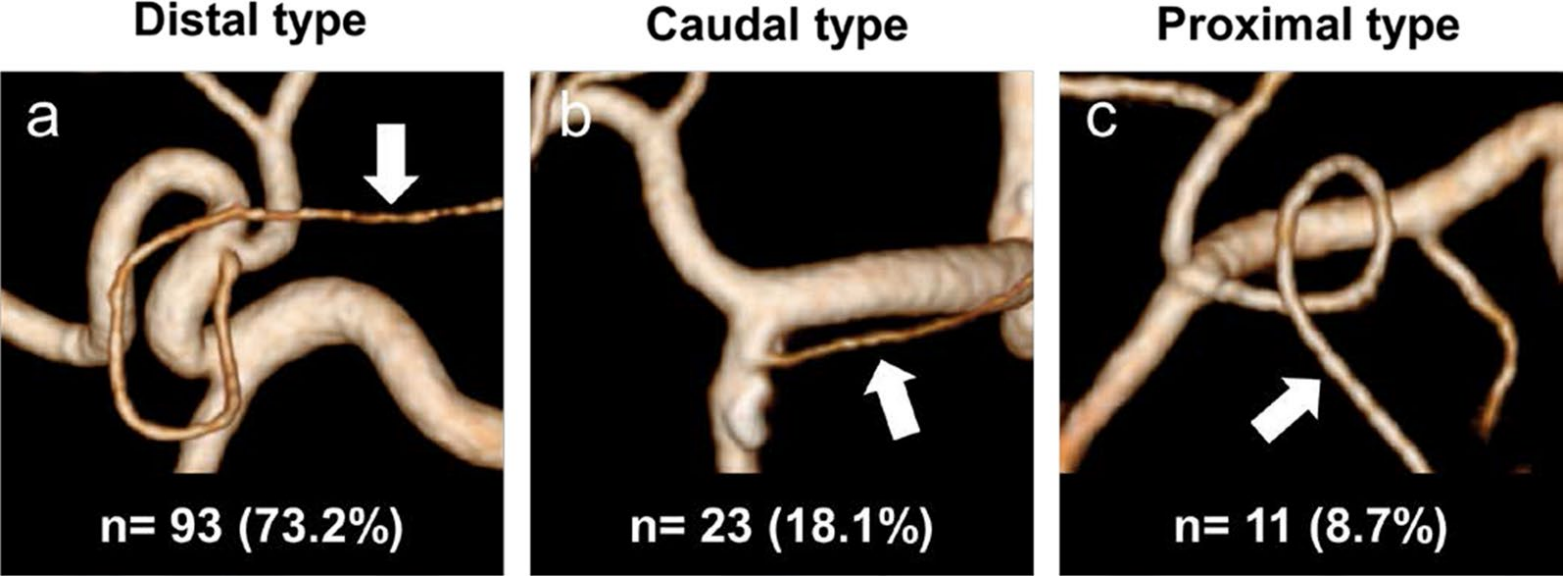
The branching patterns of the right gastric artery (RGA). **a** Distal type, RGAs branching from the proper hepatic, the right hepatic or the left hepatic arteries. **b** Caudal type, RGAs branching from the gastroduodenal artery (GDA). **c** Proximal type, RGAs branching from the common hepatic artery (CHA) or the branching point of the GDA from the CHA. *Arrows* indicate the RGA

The RGA was clearly visualized using 3D angiography in all 127 cases. The branching patterns of the RGA were classified into three types according to our previous classification: distal type, in which the RGA ramifies from the proper hepatic artery (PHA), the right hepatic artery, or the LHA; caudal type, in which the RGA ramifies from the gastroduodenal artery (GDA); and proximal type, in which the RGA ramifies from the CHA or the branching point of the GDA from the CHA [15]. Ninety-three RGAs (73.2%) were classified into distal type (Fig. 4a); 23 RGAs (18.1%), caudal type (Fig. 4b); and 11 RGAs (8.7%), proximal type (Fig. 4c).

### Running aspects of the hepatic artery relative to the PV

The hepatic arteries including the PHA, right hepatic artery and LHA were clearly visualized around the PV in all 127 cases. We classified the running aspects of the hepatic artery relative to the PV into three types: ventral to the PV (ventral type), medial to the left margin of the PV (medial type), and dorsal to the PV (dorsal type). Ninety-eight cases were classified into ventral type (77.2%, Additional file 1: Fig. S2a); 26 medial type (20.5%, Additional file 1: Fig. S2b); and 3 dorsal type (2.4%, Additional file 1: Fig. S2c).

### Branching patterns of the infra-pyloric artery (IPA)

**Fig. 5:**
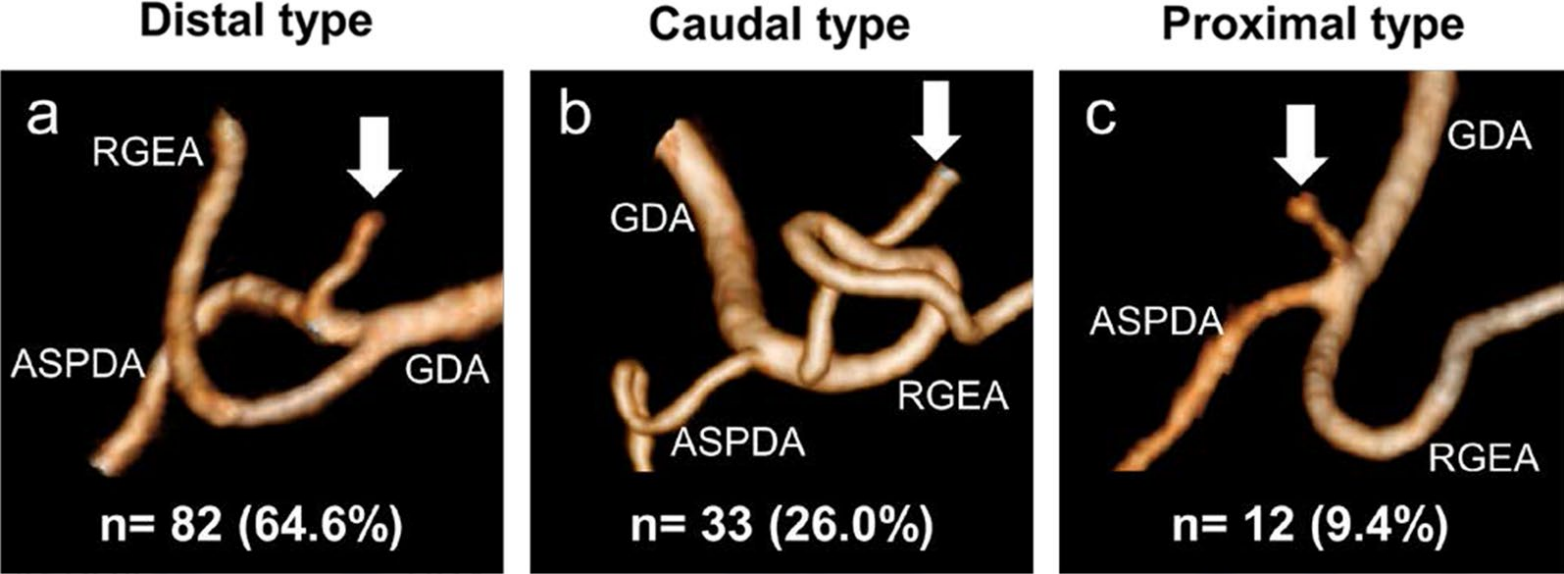
The branching patterns of the infra-pyloric artery (IPA). **a** Distal type, IPAs branching from the anterior superior pancreaticoduodenal artery (ASPDA) or the branching point of ASPDA from gastroduodenal artery (GDA). **b** Caudal type, IPAs branching from the right gastroepiploic artery. **c** Proximal type, IPAs branching from the GDA. *Arrows* indicate the IPA. RGEA, right gastroepiploic artery

The IPA was clearly visualized using 3D angiography in all 127 cases. The branching patterns of the IPA were classified into three types according to the Haruta classification: distal type, in which the IPA ramifies from the anterior superior pancreaticoduodenal artery or the branching point of the anterior superior pancreaticoduodenal artery from the GDA; caudal type, in which the IPA ramifies from the right gastroepiploic artery; and proximal type, in which the IPA ramifies from the GDA [17]. A total of 82 IPAs (64.6%) were classified into distal type (Fig. 5a), 33 (26.0%) into caudal type (Fig. 5b), and 12 (9.4%) into proximal type (Fig. 5c).

### Branching patterns of the posterior gastric artery (PGA)

**Fig. 6:**
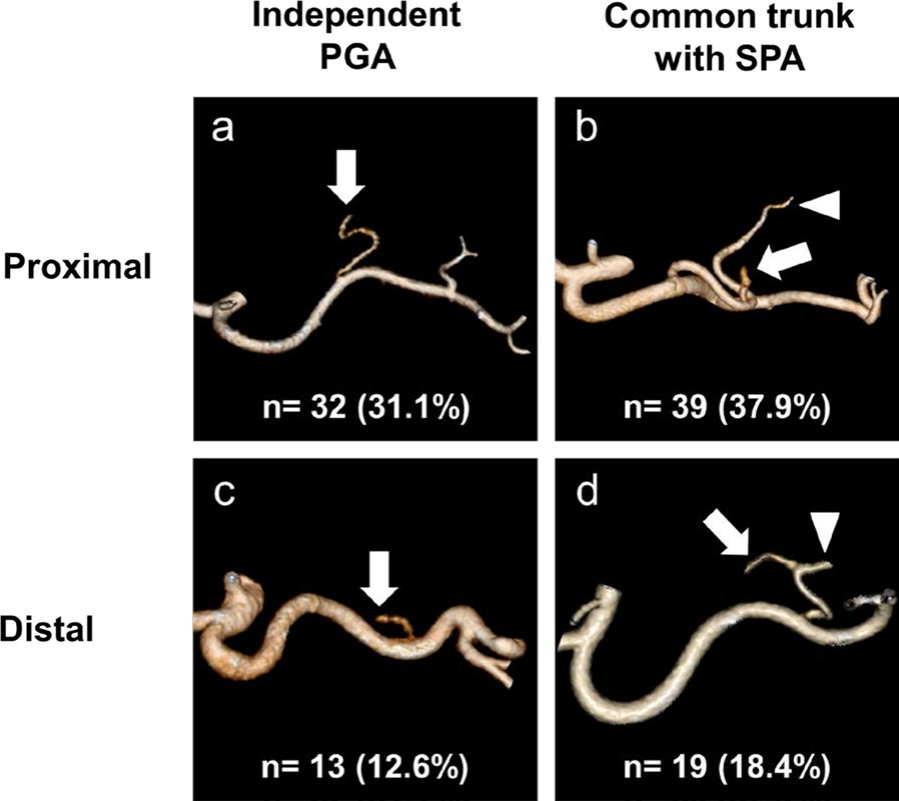
The branching patterns of the posterior gastric artery (PGA). **a** PGAs branching directly from the proximal half between the origin of the splenic artery (SA) and the pancreatic tail end. **b** PGAs branching from a common trunk with the superior polar artery (SPA) which ramifies from the proximal half between the origin of SA and the pancreatic tail end. **c** PGAs branching directly from the distal half between the origin of the SA and the pancreatic tail end. **d** PGAs branching from a common trunk with the SPA which ramifies from the distal half between the origin of SA and the pancreatic tail end. *Arrows* indicate the PGA. *Arrow heads* indicate the SPA

The PGA was clearly visualized using 3D angiography to evaluate its branching pattern in 103 cases (81.1%). We classified the PGA branching patterns from the viewpoint of lymphadenectomy during gastrectomy. The SA was divided in half from its origin to the pancreatic tail end according to the Japanese classification of gastric carcinoma [18]. Thirty-two PGAs (31.1%) ramified independently from the proximal half between the origin of the SA and the pancreatic tail end (Prox-I; Fig. 6a). In 39 cases (37.9%), a common trunk of the PGA and the superior polar artery (SPA) ramified from the proximal half between the origin of the SA and the pancreatic tail end (Prox-C; Fig. 6b). Thirteen PGAs (12.6%) ramified independently from the distal half between the origin of the SA and the pancreatic tail end (Dist-I; Fig. 6c). In 19 cases (18.4%), a common trunk of the PGA and SPA ramified from the distal half between the origin of the SA and the pancreatic tail end (Dist-C; Fig. 6d). Notably, the SPA ramified independently from the proximal and distal half between the origin of the SA and the pancreatic tail end in 3 cases (2.9%) and 9 cases (8.7%), respectively.

### Branching patterns of the left gastroepiploic artery (LGEA) and its omental branch

**Fig. 7:**
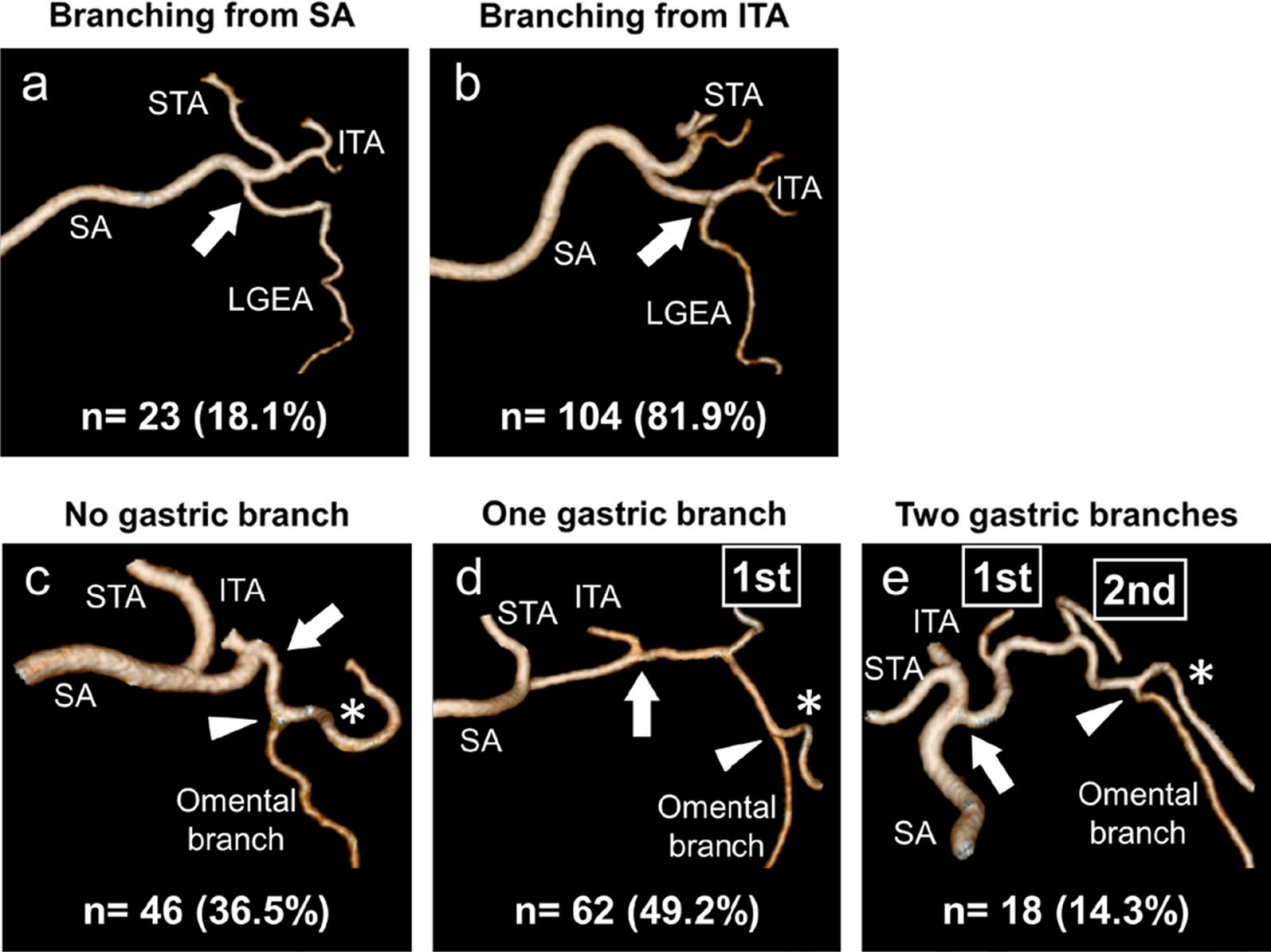
The branching patterns of the left gastroepiploic artery (LGEA) and its omental branch. **a** LGEAs branching from the splenic artery (SA). **b** LGEAs branching from the inferior terminal artery (ITA). **c** There is no gastric branch between the roots of the LGEA and its omental branch. **d** There is one gastric branch between the roots of the LGEA and its omental branch. **e** There are two gastric branches between the roots of the LGEA and its omental branch. *Arrows* indicate the roots of the LGEA. *Arrow heads* indicate the branching point of the omental branch. *LGEA distal to its omental branch. *ITA* inferior terminal artery, *SA* splenic artery, *STA* superior terminal artery

The LGEA was clearly visualized using 3D angiography in all 127 cases. The LGEA ramified from the SA in 23 cases (18.1%; Fig. 7a) and from the inferior terminal artery in 104 cases (81.9%; Fig. 7b). The omental branch of the LGEA was also visualized in 126 cases (99.2%). The branching point of the omental branch was classified into three types based on the number of gastric branches between the roots of the LGEA and its omental branch. No, one, and two gastric branches were found in 46 cases (36.5%; Fig. 7c), 62 cases (49.2%; Fig. 7d), and 18 cases (14.3%; Fig. 7e), respectively, between the roots of the LGEA and its omental branch.

## Discussion

We evaluated the vascular anatomies in detail for the CA, LGV, LGA, LIPA, RGA, IPA, PGA, and LGEA using a 3D angiography reconstructed from enhanced MDCT data. To our knowledge, this study is the first to conduct a comprehensive analysis of perigastric vessel anatomies using a 3D angiography.

Laparoscopy-assisted distal gastrectomy was first reported in 1994. It has been widely performed for early gastric cancer in recent years in Asian countries including Japan [8, 19, 20]. A series of randomized clinical trials confirmed the non-inferiority of laparoscopy-assisted distal gastrectomy to open distal gastrectomy in terms of adverse events, short-term clinical outcomes, and relapse-free and overall survivals for Stage I gastric cancer [3–7]. The non-inferiority of laparoscopic distal gastrectomy (LDG) for advanced gastric cancers was also reported by several randomized clinical trials, which suggested that experienced surgeons can safely perform LDG with D2 lymphadenectomy for advanced gastric cancer [21–24]. In contrast to the multicenter trials conducted in high-volume centers, retrospective cohort studies based on a Japanese nationwide registry database revealed a higher incidence of pancreatic fistula in LDG than in open distal gastrectomy, but wound infection and dehiscence were less common in the LDG group [25, 26]. In general practice, LDG seems to be a feasible therapeutic alternative for gastric cancer, but further improvements in surgical quality are warranted. On the contrary, the indication of laparoscopic gastrectomy has gradually increased in Asian countries. Laparoscopic total gastrectomy (LTG) and proximal gastrectomy (LPG) are more commonly performed for early and advanced gastric or esophagogastric junction cancers, which require advanced surgical skills [8, 27]. Recent advances in laparoscopic surgery and the development of robotic surgery have the potential to overcome the technical difficulties of performing LTG [28]. However, preoperative simulation including the evaluation of perigastric vessel anatomies may help surgeons safely perform LDG and LTG for gastric cancers.

We have previously reported a new system of classifying RGA ramification patterns using a preoperative 3D angiography [15]. Preoperative simulation of RGA ramification patterns is useful for performing LDG or LTG. However, more information of perigastric vessel anatomy is needed for the precise and safe LTG and LPG that have been increasingly performed in recent years [8, 27]. Therefore, in this study, we comprehensively evaluated 8 perigastric vessel anatomies for the LGV, LGA with rLHA, LIPA, RGA, IPA, PGA, LGEA, and the omental branch of the LGEA from the viewpoint based on laparoscopic gastrectomy (Table 1). In our previous study, the image resolution was not satisfactory. In some cases, we were unable to trace the blood vessels to the organs such as the stomach wall. In the present study, we improved the protocols of 3D angiography; not only the RGA but also the perigastric vessels to each organ could be traced, and more accurate data were obtained.

**Table 1:** Anatomy of perigastric vessels in 127 patients who underwent MDCT followed by gastrectomy

	Visualized vessels	Classifiable vessels	Reference anatomy	No. of classification categories	Classifications
Left gastric vein (LGV)	127 (100%)	122 (96.1%)	CHA, SA	4	Dorsal of CHA, 59 (46.5%); dorsal of SA, 8 (6.3%); ventral of CHA, 27 (21.3%); ventral of SA, 28 (22.0%)
LGA with replaced LHA	18 (14.2%)	18 (14.2%)	LGA branch	3	One LGA branch, 7 (38.9%); two LGA branches, 9 (50.0%); three LGA branches, 2 (11.1%)
Left inferior phrenic artery (LIPA)	127 (100%)	124 (97.6%)	Ao, CA, LGA	6	Ao-I, 40 (31.5%); CA-I, 52 (40.9%); LGA-I, 6 (4.7%); Ao-C, 10 (7.9%); CA-C, 15 (11.8%); LGA-C, 1 (0.8%)
Right gastric artery (RGA)	127 (100%)	127 (100%)	PHA, GDA, CHA	3	Distal type, 93 (73.2%); caudal type, 23 (18.1%); proximal type, 11 (8.7%)
Infra-pyloric artery (IPA)	127 (100%)	127 (100%)	ASPDA, RGEA, GDA	3	Distal type, 82 (64.6%); caudal type, 33 (26.0%); proximal type, 12 (9.4%)
Posterior gastric artery (PGA)	103 (81.1%)	103 (81.1%)	Pancreatic tail end, SPA	4	Prox-I, 22 (31.1%); Prox-C, 39 (37.9%); Dist-I, 13 (12.6%); Dist-C, 19 (18.4%)
Left gastroepiploic artery (LGEA)	127 (100%)	127 (100%)	SA, ITA	2	SA, 23 (18.1%); ITA, 104 (81.9%)
Gastric branch of the LGEA proximal to the omental branch	127 (100%)	126 (99.2%)	SA, ITA, omental branch of the LGEA	3	No gastric branch, 46 (36.5%); one gastric branch, 62 (49.2%); two gastric branches, 18 (14.3%)

*Ao* aorta, *ASPDA* anterior superior pancreaticoduodenal artery, *C* common trunk, *CA* celiac artery, *CHA* common hepatic artery, *Dist* distal, *GDA* gastroduodenal artery, *I* independent branch, *ITA* inferior terminal artery, *LGA* left gastric artery, *LHA* left hepatic artery, *PHA* proper hepatic artery, *Prox* proximal, *RGEA* right gastroepiploic artery, *SA* splenic artery, *SPA* superior polar artery

Kawasaki et al. classified the LGV location into five types: (i) dorsal to the CHA, (ii) ventral to the CHA, (iii) ventral to the SA, (iv) dorsal to the SA, and (v) others using an MDCT without a 3D angiography [29]. Based on this classification, Yuasa et al. examined the joining pattern of the LGV using a 3D CT angiography [30]. Our results in the running aspects of the LGV relative to the CHA and SA were consistent with those of Kawasaki et al. Notably, of the 67 cases with LGVs running along the dorsal side of the arteries, 59 cases (88.1%) were running along the dorsal side to the CHA, all of which flowed into the PV. By contrast, the joining pattern of the LGV running along the ventral side to these arteries varied and did not depend on which artery the LGV was running to.

rLHA resection can cause serious liver damage or necrosis [31, 32]. Because the rLHA does not communicate with the hepatic artery in the liver, it needs to be preserved in cases with a common trunk with the LGA. In 60% of cases with a common trunk between the rLHA and the LGA, multiple branches of LGAs require attention during gastrectomy.

Greig et al. examined the right inferior phrenic artery and the LIPA using 425 cadavers, and classified the origin of the inferior phrenic arteries into eight types [33]. According to the Japanese Gastric Cancer Association, infra-diaphragmatic lymph nodes predominantly along the LIPA are categorized as no. 19 [18]. Because the origin of the LIPA, which may branch from the LGA, is important than that of the right inferior phrenic artery in gastrectomy, we focused on the branching patterns of the LIPA. In this study, the LIPA ramified from the LGA in 5.5% of the cases.

The ramification patterns of the RGA were classified into three types in our previous study on 100 cases and re-evaluated in the present study on 127 cases [15]. The two studies showed different branching rates of distal, caudal, and proximal types (68.8% vs. 72.4%, 16.9% vs. 19.5%, and 14.3% vs. 8.1%, respectively). In the present study, we were able to trace the RGAs to the stomach wall in all cases, therefore obtaining more precise data. Because caudally or proximally ramified RGAs may cause difficulty in dissecting supra-pancreatic lymph nodes, the confirmation of RGA branching rate and the high success rate of preoperative RGA visualization in this study seem to help us perform laparoscopic gastrectomy more safely.

Shinohara et al. reported laparoscopic techniques for dissection of no. 6 infra-pyloric lymph nodes and the anatomical importance of IPA [34]. Accordingly, based on the intraoperative findings, Haruta et al. classified the origins of IPA into three types: distal (64.2%), caudal (23.1%), and proximal (12.7%) [17]. In the present study, we re-evaluated the branching types of IPA using a 3D angiography: distal (64.6%), caudal (26.0%), and proximal (9.4%), which were consistent with a previous report based on intraoperative findings [17].

Although numerous researchers reported the anatomy of PGA using cadavers, the definitions and names varied [16, 35, 36]. In the present study, we defined the branch from the SA to the posterior side of the stomach as a PGA from the viewpoint of gastrectomy with lymphadenectomy. We then analyzed the PGA anatomy according to the Japanese classification of gastric carcinoma, which defined the middle from the origin of the SA to the pancreatic tail end as the boundary between nos. 11p and 11d [18]. The PGA was clearly visualized using 3D angiography in 103 cases (81.1%). The PGA was present in the proximal region in 55.9% (n = 71) of the cases, and the PGA branched as a common trunk with the SPA in 39 cases (30.7%). Many of the proximal type PGAs were closely located at the boundary of nos. 11p and 11d, which could be used as a milestone for dissection of no. 11p lymph nodes.

The LGEA branching from around the splenic hilum is an artery with many anomalies. The branching rate from the SA itself was 26.0–36.7% in previous reports [37], but it was 18.1% in the present study. Left greater curvature lymph nodes along the LGEA distal to its first gastric branch, and those along the first gastric branch of the LGEA are categorized as no. 4sb according to the Japanese Gastric Cancer Association [18]. In laparoscopic gastrectomy for early gastric cancer, the omental branch of the LGEA may be preserved. In cases with no gastric branches between the roots of the LGEA and its omental branch (36.5%; Fig. 7c), no. 4sb lymphadenectomy can be performed with preserving the omental branch by dissecting the LGEA after the branching point of its omental branch. By contrast, in cases with gastric branches ramifying from the LGEA proximal to its omental branch (63.5%; Fig. 7d and e), one or two gastric branches need to be dissected to preserve the omental branch.

In this study, we sought to evaluate the running aspects of the SA relative to the SV using a 3D-CT angiography. In one case, the SA ran along the dorsal side to the SV, however, a larger number of cases would be needed to clarify the frequency of such rare cases.

This study has several limitations. First, the vessel anatomies identified using a 3D angiography were not completely validated during surgery. Second, the clinical values of the preoperative simulation of perigastric vessels for laparoscopic gastrectomy, such as shorter operation time and less complications, were not evaluated in clinical studies. However, our data showed detailed anatomical variations in perigastric vessels, understanding of which will likely help surgeons to perform laparoscopic surgery safely. In addition, this study revealed that a 3D angiography is potentially useful for precise visualization of small perigastric vessels that may be used for preoperative simulation as well as for education to young doctors and medical students.

## Conclusions

We re-evaluated perigastric vessel anatomies for LGV, LGA with rLHA, LIPA, RGA, IPA, PGA, LGEA, and the omental branch of the LGEA from the viewpoint based on laparoscopic gastrectomy. Preoperative 3D angiography is useful for the precise evaluation of perigastric vessel anatomies, and may help us to perform laparoscopic gastrectomy and robotic surgery safely.

## Supplementary Information

**Additional file 1: Fig. S1.** Branching patterns of the celiac artery and the left gastric artery (LGA). **Fig. S2.** Running aspects of the hepatic artery relative to the portal vein (PV).

## Data Availability

The datasets used and/or analyzed during the current study are available from the corresponding author on reasonable request.

## Abbreviations

3D: Three-dimensional
Ao: Abdominal aorta
BIPAs: Bilateral inferior phrenic arteries
CHA: Common hepatic artery
CT: Computed tomography
GDA: Gastroduodenal artery
IPA: Infra-pyloric artery
LDG: Laparoscopic distal gastrectomy
LGA: Left gastric artery
LGEA: Left gastroepiploic artery
LGV: Left gastric vein
LHA: Left hepatic artery
LIPA: Left inferior phrenic artery
MDCT: Multidetector-row computed tomography
PGA: Posterior gastric artery
PHA: Proper hepatic artery
PV: Portal vein
RGA: Right gastric artery
rLHA: Replaced left hepatic artery
SA: Splenic artery
SPA: Superior polar artery
SV: Splenic vein
