# Supplementary material for: Comprehensive evaluation of three-dimensional anatomy of perigastric vessels using enhanced multidetector-row computed tomography
Source: BMC Surg. 2022 Nov 21;22:403. doi: 10.1186/s12893-022-01836-0 (PMC9677658; doi:10.1186/s12893-022-01836-0)
Supplement: Supplementary file 1 — Additional file 1: Fig. S1. Branching patterns of the celiac artery and the left gastric artery (LGA). Fig. S2. Running aspects of the hepatic artery relative to the portal vein (PV). [file 12893_2022_1836_MOESM1_ESM.pdf]

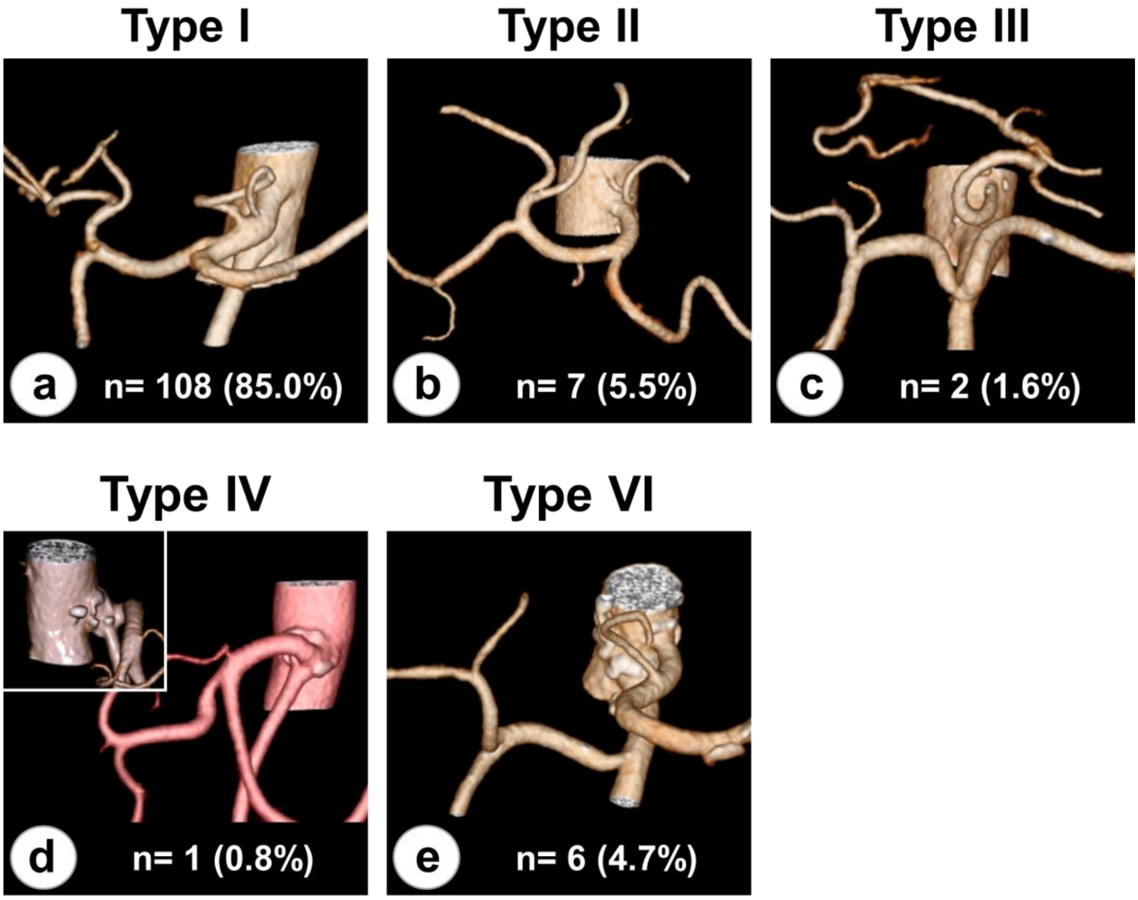

Fig. S1. Branching patterns of the celiac artery and the left gastric artery (LGA). (a) Type I, common trunk of LGA, splenic artery (SA), and common hepatic artery (CHA). (b) Type II, common trunk of SA and CHA. (c) Type III, common trunk of CHA, SA, and the superior mesenteric artery (SMA). (d) Type IV, common trunk of LGA, SA, CHA, and SMA. (e) Type VI, common trunk of LGA and SA, and CHA branched from SMA.

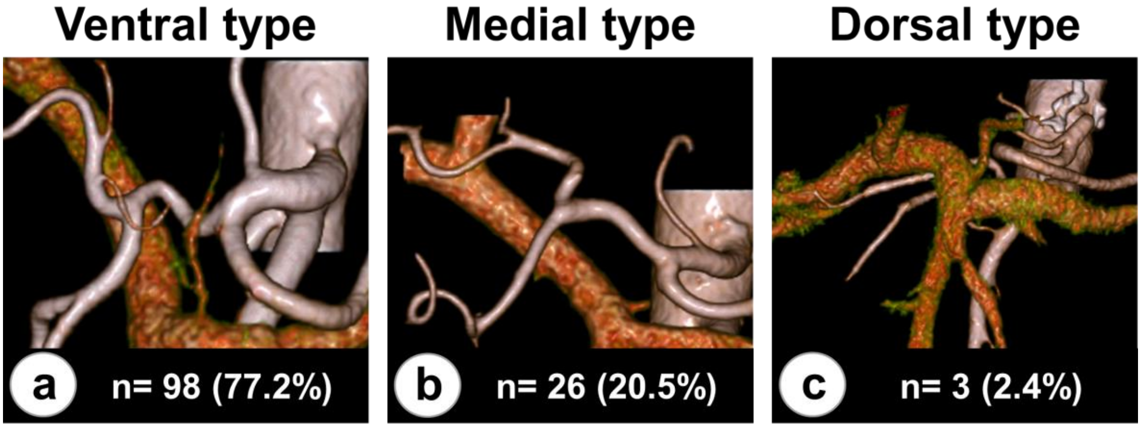

Fig. S2. Running aspects of the hepatic artery relative to the portal vein (PV).

(a) Ventral type, hepatic artery running ventral to the PV. (b) Medial type, hepatic artery running medial to the left margin of the PV. (c) Dorsal type, hepatic artery running dorsal to the PV.
